# Morphological study of the supratrochlear foramen in *Canis lupus* ssp.

**DOI:** 10.1111/joa.70153

**Published:** 2026-04-15

**Authors:** Mariana Batista, Maria Soares, Eduardo Marcelino, Luísa Mendes‐Jorge, Graça Alexandre‐Pires, Cleia Detry, Sónia Gabriel, Dulce Ferreira, Ana Elisabete Pires, Joana Catita

**Affiliations:** ^1^ Research in Veterinary Medicine, I‐MVET, Faculty of Veterinary Medicine Lusófona University‐Lisbon University Centre Lisbon Portugal; ^2^ Egas Moniz Center for Interdisciplinary Research (CiiEM) Egas Moniz School of Health & Science Caparica Portugal; ^3^ Centre for Interdisciplinary Research in Animal Health (CIISA), Faculty of Veterinary Medicine University of Lisbon Lisbon Portugal; ^4^ Associate Laboratory for Animal and Veterinary Sciences (AL4AnimalS) Lisbon Portugal; ^5^ Archaeology Center of the University of Lisbon Lisbon Portugal; ^6^ Archaeosciences Laboratory (LARC), Cultural Heritage, P.I. (PC, I.P.) Lisbon Portugal; ^7^ Research Centre in Biodiversity and Genetic Resources (BIOPOLIS‐CIBIO‐InBIO) University of Porto Vairão Portugal; ^8^ CECAV ‐ Animal and Veterinary Research Center, Faculty of Veterinary Medicine (FMV) Lusófona University Lisbon Portugal

**Keywords:** comparative osteometry, domestic dog, evolutionary anatomy, humeral morphology, supratrochlear foramen (STF)

## Abstract

The domestic dog (*Canis lupus familiaris*), a species characterized by remarkable phenotypic diversity resulting from selective breeding, is among the several mammals that may exhibit an anatomical perforation of the humeral condyle, designated as the supratrochlear foramen (STF). The prevalence of the STF varies considerably across mammalian species and has been proposed to reflect functional adaptations linked to evolutionary and biomechanical factors, particularly in the human humerus. To the best of our knowledge, comprehensive studies on STF variation within present‐day canine populations remain scarce. This study aimed to determine the prevalence and morphology of the STF in extant dogs, and to compare these findings with those from Roman Imperial Period dogs, and Iberian wolves (*Canis lupus signatus*), a subspecies considered representative of the dog's wild ancestor, in order to explore evolutionary changes within *Canis lupus* subspecies. We analyzed 269 humeri (123 extant dogs, 76 ancient dogs, 70 Iberian wolves) using osteometric measurements and statistical analysis. Our results demonstrate that the STF was observed in 73.17% of extant dogs, compared to 82.89% in ancient dogs and 98.57% in Iberian wolves, suggesting a reduction in its prevalence over time in domestic lineages that may be linked to human‐driven selection processes. In all studied groups, the STF was consistently located closer to the lateral humeral epicondyle, and its predominantly transversely elongated shape reveals a common morphological pattern across *Canis lupus* subspecies. Despite differences in humeral size among groups, STF dimensions showed a positive correlation with overall humeral measurements, with larger humeri exhibiting proportionally larger and more frequent STFs. Extant dogs displayed the smallest STF dimensions, followed by ancient dogs, with Iberian wolves having the largest. Considering the potential impact of the STF on the biomechanical properties of the distal humerus, this study provides new insights into its anatomical variability in dogs, emphasizing its clinical relevance for orthopedic diagnostic accuracy and surgical treatments in veterinary medicine.

## Graphical abstract

The supratrochlear foramen (STF) is a common anatomical feature in the humeri of dogs and wolves. This study shows that the STF exhibits a consistent shape and position in dogs and wolves, suggesting a common morphological pattern; however, it was less frequent in smaller dog humeri, and its size correlated with humeral dimensions, suggesting that humeral size plays a key role in this variation.

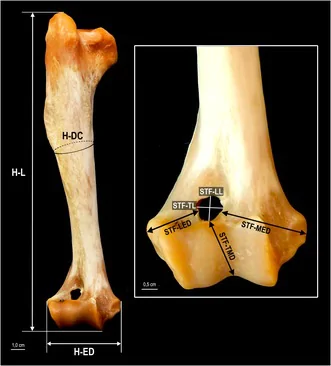

## 1 INTRODUCTION

Since the Roman period, about 2000 years ago, domestic dogs (*Canis lupus familiaris*) have undergone highly selective artificial selection (Benecke, 1987) for phenotypical characteristics and traits, including the improvement of their working abilities as assistants in human rural activities, companionship for emotional purposes or simply for altered aesthetics (Horard‐Herbin et al., 2014; MacKinnon, 2010; Pires et al., 2019). As a result, extant dogs display a remarkable range of phenotypic diversity, which, among others, includes morphological variations in bone size and shape (Bannasch et al., 2020; Darwin, 1859; Shearin & Ostrander, 2010). Such diversity has spurred continued scientific interest in understanding canine skeletal morphological variation, prompting ongoing research using traditional osteometric analyses (Nganvongpanit et al., 2017; Toryan et al., 2024; Toth & Siegel, 2021) as well as more modern methods such as computed tomography‐based approaches that provide highly precise data (Al Aiyan et al., 2019; Al Aiyan et al., 2021).

The canine humerus presents a perforation on the thin bone plate of the articular condyle (*Condylus humeri*) that separates the radial (*Fossa radialis*) and olecranon fossae (*Fossa olecrani*) (Barone, 2020; Sisson & Grossman, 1977). The *Nomina Anatomica Veterinaria* (ICVGAN, 2017) and anatomical textbooks refer to this structural feature as the supratrochlear foramen (STF) (*Foramen supratrochleare*), but contrary to most foramina (Shivaleela et al., 2016), no anatomical structures pass through this opening (Barone, 2020; Evans & de Lahunta, 2013). This foramen was first described in humans during the early nineteenth century by Meckel (1816). Since then, an abundance of nomenclature has been used to describe it, including olecranon foramen (Lamb, 1890), septal aperture (Hrdlicka, 1932), supracondylar foramen (Fearnside et al., 2002), intercondylar foramen (Kumarasamy et al., 2011), supratrochlear aperture (Ndou, 2016), and olecranon aperture (Pires et al., 2019). Such terminological inconsistency in literature may compromise the reliability of comparative analysis studies (Strzelec et al., 2017).

The STF is found exclusively in mammals, both in the lower and in the higher orders, while it is absent in swimming, flying, and terrestrial species adapted to life in water (Hirsch, 1928), showing considerable variability across species, and among single individuals (Hirsh, 1927; Lamb, 1890; Meckel, 1816). Among terrestrial mammals, some carnivores, including dogs and wolves, exhibit this feature (Hirsh, 1927; Lamb, 1890; Meckel, 1816). Early studies by Lamb (1890) reported that the STF in dogs is consistently present and relatively large, enabling full extension of the elbow joint during feeding and pulling, thereby indicating that such movement would not be possible without this foramen. This supports the hypothesis that repetitive mechanical stress related to limb use and positioning plays a key role in the development of the STF (De Wilde et al., 2004; Hirsh, 1927; Kubicka et al., 2015; Lamb, 1890). However, there is still uncertainty regarding the exact cause of STF formation across species (Baker et al., 2022). Factors such as bone robusticity (Benfer & McKern, 1966; Ndou & Schepartz, 2016), dietary influences on bone metabolism, and the reabsorption of the bony septum between the fossae after humeral condyle ossification (Mays, 2008) have been hypothesized to contribute to STF development (Bradshaw et al., 2020). Additional findings indicate that humeri with an STF display a shorter and narrower distal medullary canal (Paraskevas et al., 2010), although a more recent study found no correlation between STF presence and medullary canal width (Ndou et al., 2017).

Beyond the study of its inception as an indicator of bone biomechanical or metabolic adaptations, this anatomical variation has also been recognized as clinically significant, specifically in improving orthopedic diagnostic accuracy (Erdogmus et al., 2014) and surgical treatment of canine supracondylar fractures (Fearnside et al., 2002; Matthiesen, 1992), which frequently extend across the STF (DeCamp et al., 2016). Supracondylar fractures in dogs are technically challenging to repair (Guerin et al., 1998; Klause et al., 1990) largely due to limited bone available for plate fixation or screw and pin placement (Guerin et al., 1998; Klause et al., 1990), further compounded by the presence of the STF (Matthiesen, 1992), as well as the irregular surface of the humeral distal epiphysis and risk of elbow range motion compromise (Fearnside et al., 2002; Guerin et al., 1998; Klause et al., 1990; Perry et al., 2015). Additionally, the existence of the foramen may also increase susceptibility to low‐energy fractures of the distal humerus (Sahajpal & Pichora, 2006).

Despite extensive research, the characterization of STF's morphology and its clinical significance in dogs remains scarce, highlighting the need for further investigation. Taking the extraordinary phenotypic variation of the present‐day canine population into consideration, this study aims to determine the STF prevalence and explore its morphology in domestic dogs while comparing it to humeri from historical domestic dogs (dated to the Roman Imperial Period) and Iberian wolves (*Canis lupus signatus*) as a proxy to the ancestral species of the dog for a better understanding of its evolutionary changes among the *Canis lupus* species and subspecies. This knowledge holds relevance not only for academic purposes but also to improve clinical practices, particularly regarding accurate interpretation of diagnostic imaging and the development of better‐fitting surgical techniques in veterinary medicine.

## 2 METHODS

### 2.1 Dog and Iberian wolf specimens

Bone collections from the Faculty of Veterinary Medicine of Lusófona University, the Faculty of Veterinary Medicine of Lisbon University, the National Museum of Natural History and Science in Lisbon, the Archaeosciences Laboratory (LARC) and The Centre for Archaeology of the University of Lisbon (UNIARQ) were analyzed for this study, comprising a total of 269 humeri (Table 1). Of these, 123 were from extant Portuguese dogs, including 40 confirmed left–right humeri pairs from the same animal, 76 from Iberian archeological dog specimens, dated from the Roman Imperial Period, 1st century AD from a ritual context at Calle Almendralejo in Mérida, Spain, the capital of Lusitania—the Roman province of southwestern Iberia (Detry et al., 2024), and 70 from extant Iberian wolves, including 32 confirmed left–right humeri pairs from the same animal. For the remaining specimens, information on laterality and individual association was not available. With few exceptions, breeds, age and sex were unknown for all specimens. Juvenile humeri with incomplete bone ossification or bones with pathological alterations were excluded from the study. Whenever an accurate osteometric measurement was possible for a given humerus, despite other bone abnormalities or taphonomy losses, it was included in the study, and, as such, sample size (*n*) for each variable varies accordingly.

**TABLE 1:** Humeri sample description (*n* = 269).

*Species*	Humeri (*n*)		Proximal epiphysis integrity (*n*)
*Canis lupus familiaris*			
Extant	123	L (58) R (65)	Intact (123)
Roman period	76	L (41) R (35)	Intact (33) Partial^a^ (43)
*Canis lupus signatus*	70	L (33) R (37)	Intact (70)

Abbreviations: L, Left; R, right.

^a^ Partial bone integrity due to fractures, preservation or taphonomy loss.

### 2.2 Osteometry

All humeri underwent similar systematic osteometric analysis to ascertain their dimensions in fixed anatomical landmarks, as represented in Figure 1. Namely, maximum humerus length (H‐L) was determined by measuring the distance between the more proximal end of the major trochanter and the more distal end of the trochlea, and humeral diaphyseal circumference (H‐DC) was measured immediately below the distal end of the deltoid tuberosity using a ruler and a measuring tape, respectively, by a single observer. In the distal epiphysis of the humerus, the maximum distance between the lateral epicondyle and the medial epicondyle (H‐ED) was measured using a digital caliper (range 0–150 mm ±0.1 mm; PEREL®, Portugal) by two independent observers, with each measurement performed in duplicate by each observer.

**FIGURE 1:**
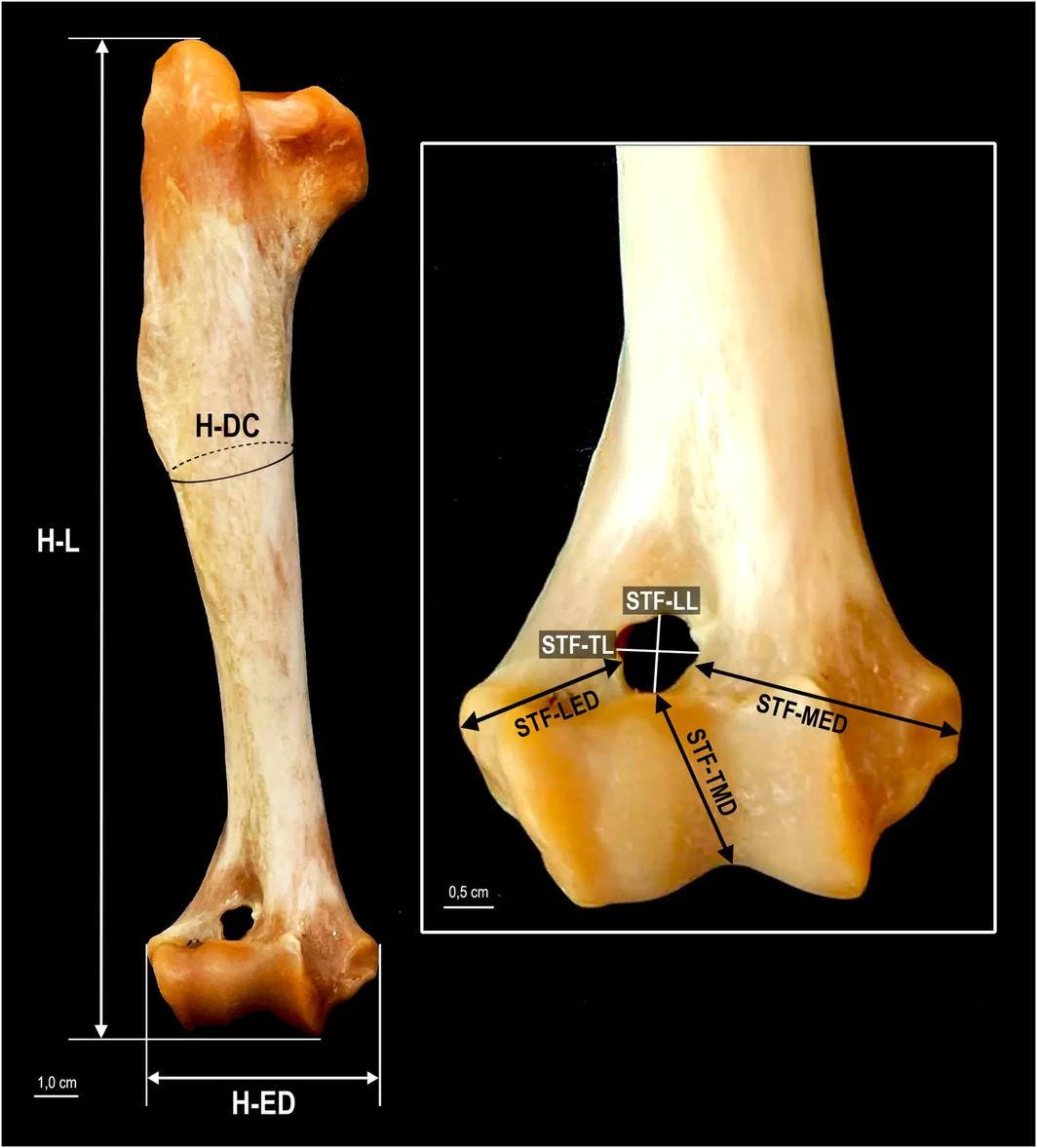
Illustration of the osteometric measurements taken of a humerus with and supratrochlear foramen (STF), shown in a detailed close‐up. A cranial view of the right humerus of an extant dog was used for representation. H‐DC, humeral diaphyseal circumference; H‐ED, maximum epicondyle distance; H‐L, maximum humerus length; STF‐LED, STF distance to the lateral epicondyle; STF‐LL, STF longitudinal length; STF‐MED, STF distance to the medial epicondyle; STF‐TL, STF transverse length; STF‐TMD: STF distance to the trochlear margin. Scale bars (right): 1.0 cm; (left): 0.5 cm.

STF prevalence was established by assessing the presence or absence of an aperture on the humeral condyle. Whenever a clear perforation was not discernible, the presence of a translucent septum was investigated by holding a light against the olecranon fossa, as described by Mathew et al. (2016) (Figure 3a). Two independent observers performed osteometric analysis of each STF and each measurement was taken in duplicate using digital calipers (range 0–150 mm ±0.1 mm; PEREL®, Portugal). The longitudinal (STF‐LL) and transverse length (STF‐TL) of the foramen, as well as its distance from the lateral epicondyle (STF‐LED), the medial epicondyle (STF‐MED) and the trochlear margin (STF‐TMD) were recorded. The ratio between STF‐LL and STF‐TL was calculated to classify the STF as having a longitudinal (LL/TL <0.95) or a transverse shape (LL/TL >1.05).

### 2.3 Body size estimation and classification of extant dog specimens

To improve result interpretation, shoulder height estimations were performed from humeri osteometric measurements of extant dogs, followed by categorization of specimens into three body size categories (small, medium, and large). Shoulder height estimation was based on H‐L (cm), applying the formula developed by Koudelka (1885): Shoulder Height (cm) = 3.37 × H‐L (cm), with small dogs having less than 40 cm, medium dogs between 40 and 60 cm, and large dogs having over 60 cm of shoulder height. However, it should be noted that these categories refer only to one osteometric measurement (H‐L) and dog precise shoulder height is therefore only estimated and subjected to a considerable margin of error.

### 2.4 Statistical analysis

The obtained data were compiled and analyzed using IBM SPSS Statistics (Version 29.0). Descriptive statistics (mean, maximum and minimum values, and standard error of the mean) were obtained for all measurements and indexes. Normality tests (Shapiro–Wilk) were performed on all variables. To investigate bone symmetry either paired *t*‐tests or Wilcox tests were performed whether the variables were normally distributed or not, respectively. Overall osteometric differences between present‐day dogs and Iberian wolves and between present‐day dogs and ancient dogs were investigated using *t*‐test for normally distributed variables and the non‐parametrical Mann–Whitney *U* test for non‐parametrically distributed data. Correlations among relevant measurements were analyzed using the Spearman test. STF prevalence was analyzed using chi‐squared tests. All data are presented as mean ± standard error of the mean. Differences between groups were considered statistically significant when the *p* < 0.05. Exact p‐values are reported unless they are smaller than 0.001, in which case they are presented as *p* < 0.001.

## 3 RESULTS

### 3.1 Dogs and Iberian wolves' humeri analysis

The analysis was initially performed on the available humeri pairs of extant dogs and Iberian wolves, to investigate potential asymmetries. Data showed no significant asymmetries in extant dogs' humeri regarding overall size measurements (H‐L, H‐DC, and H‐ED), STF dimensions (STF‐LL and STF‐TL) or STF placing within the humerus (STF‐LED, STF‐MED, and STF‐TMD). Conversely, a slight but statistically significant asymmetry was found in Iberian wolves' humeri, as the left humeri were longer (20.50 cm vs. 20.41 cm; *p* = 0.008), thinner (6.22 cm vs. 6.31 cm; *p* = 0.023) and with a shorter lateral to medial epicondyle distance (42.78 cm vs. 43.1 cm; *p* = 0.011) than their right counterparts. Additionally, left STF extended further to the medial epicondyle, as the STF‐MED was smaller in the left than in the right humeri (23.39 cm vs. 23.86 cm; *p* = 0.001). Further analysis was conducted considering individual humeri.

Osteometric analysis of the humerus in extant dogs revealed a mean H‐L of 12.93 ± 0.41 cm, ranging from 7.5 to 25.0 cm; a H‐DC of 4.49 ± 0.14 cm, with values between 2.3 and 8.2 cm; and a mean H‐ED of 29.52 ± 0.80 mm, with a minimum of 15.7 and a maximum of 51.5 mm (Table 2). In ancient dogs, the mean values for H‐L, H‐DC, and H‐ED were similar to those observed in extant dogs (*p* = 0.722, *p* = 0.864 and *p* = 0.731, respectively). In comparison, the Iberian wolf exhibited the largest mean values, showing a H‐L of 20.43 ± 0.15 cm, H‐DC of 6.27 ± 0.06 cm, and H‐ED of 42.87 ± 0.35 mm. As expected, all Iberian wolf humeri measurements were more consistent, presenting a smaller variability, compared to extant dogs (Table 2).

**TABLE 2:** Humerus dimensions of dog and Iberian wolf.

	H‐L (cm)			H‐DC (cm)			H‐ED (mm)		
	Mean ± SE (±SD)	Max	Min	Mean ± SE (±SD)	Max	Min	Mean ± SE (±SD)	Max	Min
Extant dog	12.93 ± 0.41 (±4.55) (*n* = 123)	25.0	7.5	4.49 ± 0.14 (±1.58) (*n* = 123)	8.2	2.3	29.52 ± 0.80 (±8.90) (*n* = 123)	51.5	15.7
Ancient dog	12.48 ± 0.54 (±3.18) (*n* = 35)	18.3	6.0	4.35 ± 0.17 (±1.06) (*n* = 38)	8.4	2.5	27.74 ± 0.70 (±5.45) (*n* = 61)	39.8	15.6
Iberian wolf	20.43 ± 0.15 (±1.22) (*n* = 70)	22.8	18.0	6.27 ± 0.06 (±0.46) (*n* = 70)	7.0	5.3	42.87 ± 0.35 (±2.94) (*n* = 69)	48.8	37.2

Abbreviations: H‐DC, humeral diaphyseal circumference; H‐ED, humeral epicondyle distance; H‐ML, humerus length; Min, minimum; Max, maximum; SD, standard deviation; SE, standard error.

### 3.2 Dogs and Iberian wolves' STF analysis

STF prevalence was remarkably high among Iberian wolves, reaching 98.57% (69/70), with only a single humerus lacking a STF. Ancient dogs exhibited a slightly lower prevalence of 82.89% (63/76), while extant canine humeri only showed a prevalence of 73.17% (90/123) (Table 3). Concerning the side of the humeri, STF was present in 77.1% (37/48) of left humeri and in 79.2% (38/48) of right humeri, showing that this anatomical feature is equally likely to be found on each side (*p* = 0.805). Considering humeri pairs, we found that only 12.5% (5/40) presented an unilaterally STF, indicating that the presence of an STF in one humerus strongly indicates its presence in its counterpart (*p* < 0.001). All humeri without an STF showed a translucent septum in its place.

**TABLE 3:** STF prevalence in dog and Iberian wolf humeri.

	Present	Absent	Total	Frequency (%)
Extant dog	90	33	123	73.17
Ancient dog	63	13	76	82.89
Iberian wolf	69	1	70	98.57

In both extant and ancient dogs, as well as in wolves, the location of the STF within the distal epiphysis was significantly closer to the lateral epicondyle than to the medial epicondyle (*p <* 0.001 for comparisons between populations). The shorter distance of STF to the bone landmarks analyzed was STF‐LED, followed by STF‐TMD, and lastly STF‐MED, as described in Table 4. Extant and ancient dogs showed similar mean values for STF‐LED, STF‐TMD, and STF‐MED. Concurrently, Iberian wolves showed higher mean values, with the STF‐LED mean (13.96 ± 0.17 mm) notably shorter than the STF‐MED mean (23.63 ± 0.26 mm), and the STF‐TMD mean of 16.89 ± 0.19 mm.

**TABLE 4:** STF relative position within the distal epiphysis in dog and Iberian wolf.

	STF‐LED	STF‐MED	STF‐LED vs. STF‐MED	STF‐TMD
	Mean ± SE (±SD) (mm)	Mean ± SE (±SD) (mm)		Mean ± SE (±SD) (mm)
Extant dog	11.80 ± 0.35 (±3.34) (*n* = 90)	17.96 ± 0.57 (±5.31) (*n* = 88)	*p* < 0.001*	12.12 ± 0.37 (±3.50) (*n* = 90)
Ancient dog	10.22 ± 0.26 (±1.89) (*n* = 52)	15.73 ± 0.37 (±2.64) (*n* = 51)	*p <* 0.001*	11.36 ± 0.27 (±1.95) (*n* = 54)
Iberian wolf	13.96 ± 0.17 (±1.46) (*n* = 69)	23.63 ± 0.26 (±2.15) (*n* = 69)	*p* < 0.001*	16.89 ± 0.19 (±1.57) (*n* = 69)

Abbreviations: SD, standard deviation; SE, standard error; STF‐LED, STF distance to the lateral epicondyle; STF‐MED, STF distance to the medial epicondyle; STF‐TMD, STF distance to the trochlear margin; vs., versus.

* Student *t*‐test significant difference between groups (*p* < 0.05).

A wide range of STF shapes and sizes was observed in all three populations. In extant dogs, the mean STF‐LL and STF‐TL values were 5.12 ± 0.27 mm and 6.00 ± 0.28 mm, respectively (Figure 2). Ancient dogs, although humeri size was similar to the size of extant dogs, had significantly larger STF, with STF‐LL and STF‐TL measuring 6.02 ± 0.30 mm and 7.04 ± 0.30 mm (*p* = 0.003 and *p* = 0.005, respectively). Finally, and according to their overall larger humeri dimensions, the Iberian wolf showed the largest STF when compared to both extant and ancient dogs, with mean STF‐LL and STF‐TL values of 9.90 ± 0.23 mm and 10.73 ± 0.19 mm (*p* < 0.001 for all comparisons between populations). Based on STF‐LL/STF‐TL ratio, the STF was predominantly more transverse (ratio <0.95), or even more equally proportionally shaped (ratio 0.95–1.05 mm) than predominantly longitudinal (Table 5).

**FIGURE 2:**
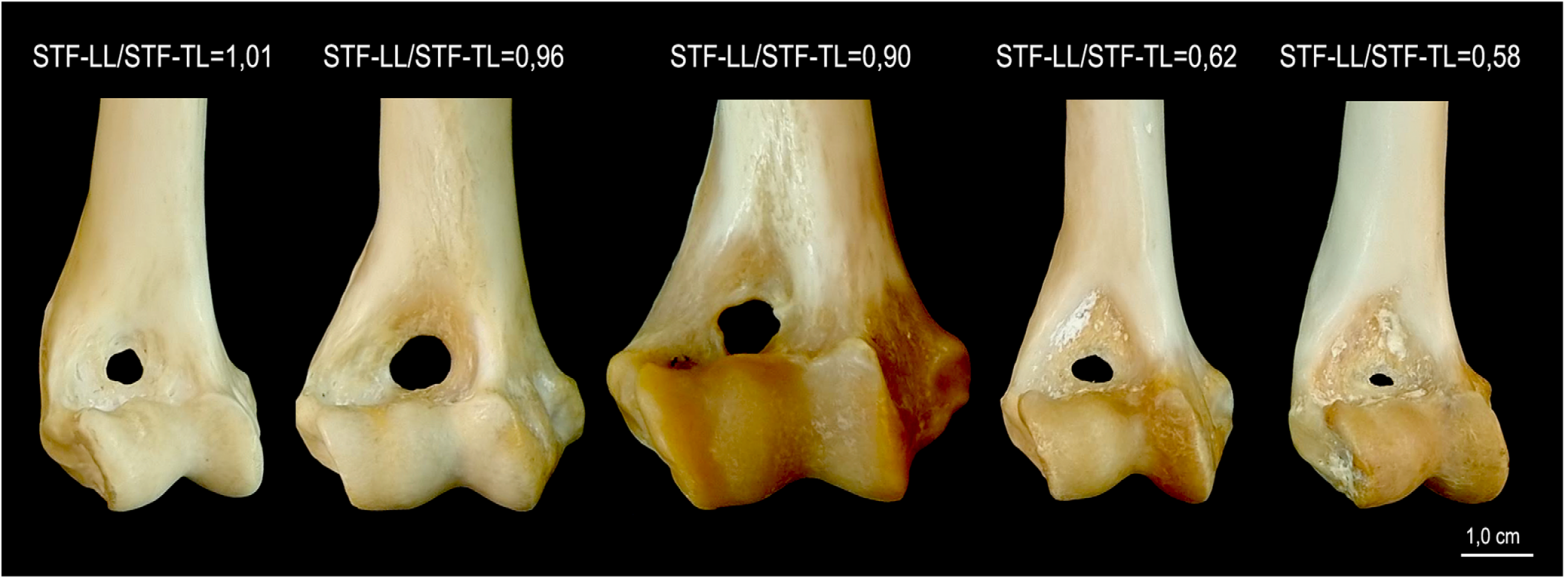
STF diversity on size and shape of humeri from extant dogs (cranial view). Scale bar (A): 1.0 cm.

**TABLE 5:** STF dimensions in dog and Iberian wolf humeri.

	STF‐LL	STF‐TL	STF‐LL/STF‐TL ratio		
	Mean ± SE (±SD) (mm)	Mean ± SE (±SD) (mm)	<0.95	0.95–1.05	>1.05
Extant dog	5.12 ± 0.27 (±2.50) (*n* = 90)	6.00 ± 0.28 (±2.66) (*n* = 90)	73 (81.11%)	14 (15.56%)	3 (3.33%)
Ancient dog	6.02 ± 0.30 (±2.35) (*n* = 63)	7.04 ± 0.30 (±2.38) (*n* = 63)	49 (77.78%)	14 (22.22%)	–
Iberian wolf	9.90 ± 0.23 (±1.94) (*n* = 69)	10.73 ± 0.19 (±1.55) (*n* = 69)	43 (62.32%)	20 (28.99%)	6 (8.70%)

Abbreviations: SD, Standard deviation; SE, Standard error; STF‐LL, STF longitudinal length; STF‐TL, STF transverse length.

### 3.3 Correlations between the STF and humeral dimensions in extant dogs

We further investigated whether the diversity of humeral dimensions (H‐L, H‐DC, and H‐ED) observed in extant dog correlates with variety of the size of the STF (ST‐LL and ST‐TL) or its prevalence. Statistical analysis revealed a strong positive correlation between the size of the STF and overall humeral dimensions, including total humeral length (H‐L: ρ = 0.74; *p* < 0.001), diaphyseal circumference (H‐DC: ρ = 0.628; *p* < 0.001), and epicondylar distance (H‐ED: ρ = 0.695; *p* < 0.001). These results indicate that larger humeri display proportionally larger STF. Moreover, humeri without the STF were significantly smaller than those with the STF, regarding humeral length (H‐L: *p* < 0.001), diaphyseal circumference (H‐DC: *p* < 0.001), and epicondylar distance (H‐ED: *p* < 0.001) (Figure 3). The STF was markedly less frequent in smaller humeri (i.e., below the median values for H‐L, H‐DC, and H‐ED) and significantly more frequent in larger humeri, as confirmed by chi‐squared analysis (*p* < 0.001).

**FIGURE 3:**
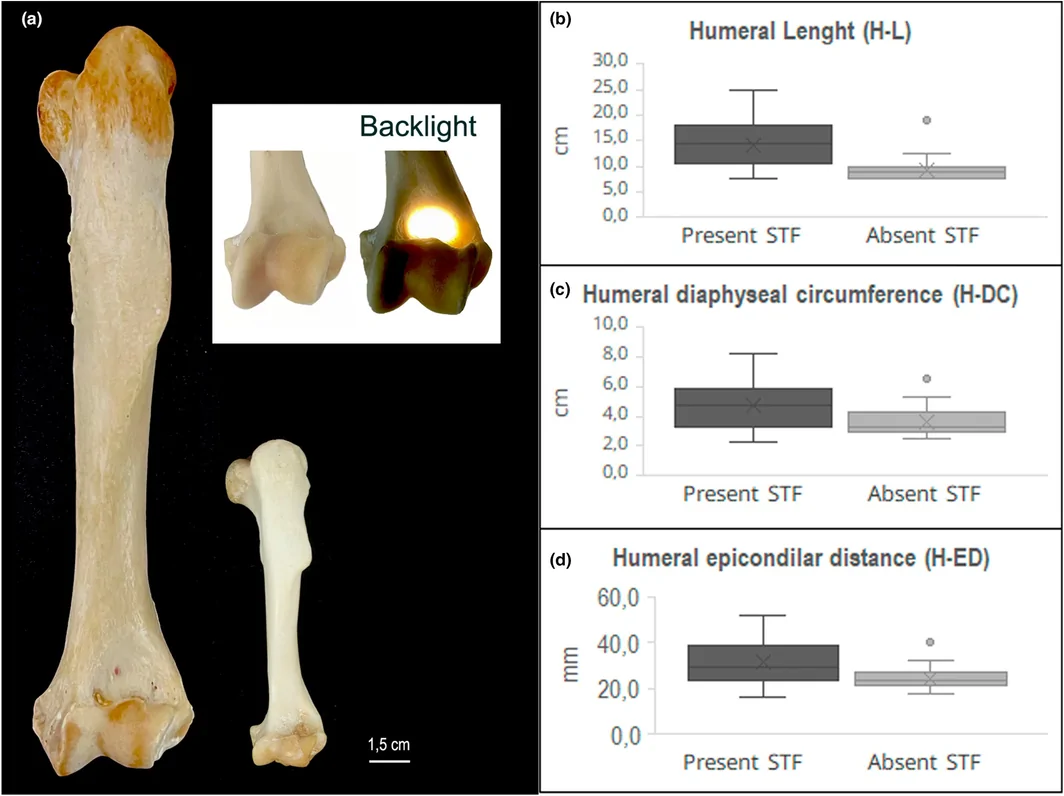
Extant dog humerus without STF (cranial view) (a) and the correlation between the presence or absence of the STF with humeri dimensions (b–d). Scale bar: 1.5 cm.

In light of the observed correlation, an additional analysis of the presence or absence of the STF according to the defined body size estimation categories (small, medium, and large dogs) was performed, as illustrated in Figure 4. As such, 67 humeri were included in the small dog category, 32 in the medium category, and 24 in the large category. Chi‐squared analysis determined that medium and large sized dogs have a statistically significant higher STF prevalence (94% and 96%, respectively) than small dogs (55%, *p* < 0.001). STF prevalence was similar between medium and larger dogs (*p* = 0.719).

**FIGURE 4:**
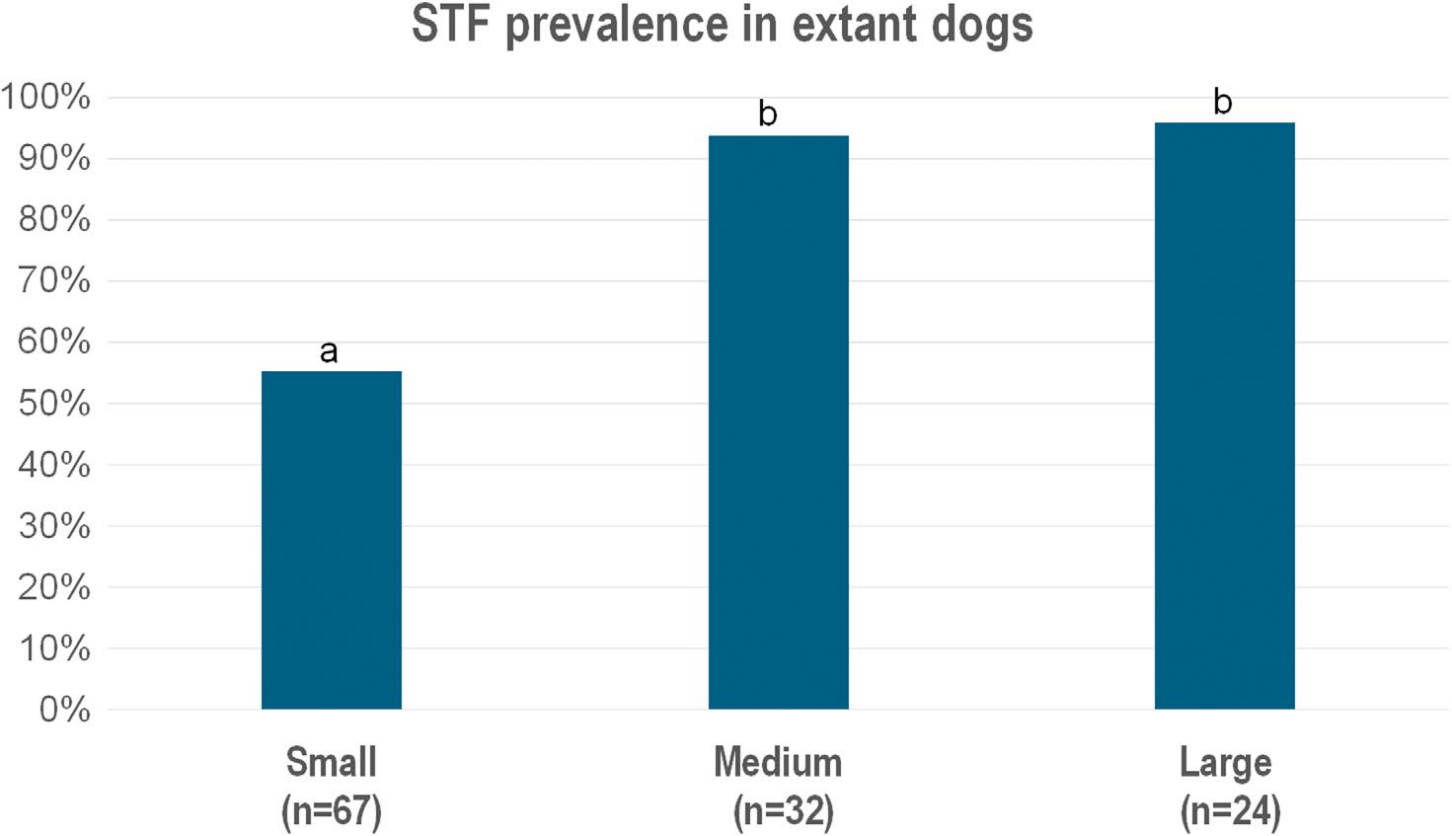
Variation in STF prevalence among extant dogs of different estimated shoulder heights (SH): Small (SH < 40 cm), medium (SH = 40–60 cm), and large (SH > 60 cm). Bars with different letters differ significantly (chi‐square; *p* < 0.05).

## 4 DISCUSSION

In the present study, osteometric analysis of the humerus evidenced that domestic dogs, both ancient and extant generally possess smaller humeri than the Iberian wolf, exhibiting a broader range of variation across all humeral dimensions: H‐L ranging from 7.5 to 25.0 cm, H‐DC from 2.3 to 8.2 cm, and H‐ED from 15.7 to 51.5 mm (Table 2). These values reflect the remarkable phenotypic diversity in humeral length, diaphyseal thickness and distal epiphysis width, existent in present‐day dog populations. The results support previous research, suggesting that selective breeding tends to increase dog's skeletal variability, allowing the emergence of both very small and very large breeds (Casinos et al., 1986; Morey, 1992; Wayne, 1986). Similarly, other studies have reported that dog's shoulder heights may range from approximately 15 cm to over 80 cm across breeds. (Bannasch et al., 2020; Bennett et al., 2016). In contrast, non‐domesticated species, such as the Iberian wolf, whose shoulder heights ranges from 70 to 80 cm (Torres & Fonseca, 2016), tend to maintain a fixed set of morphological characteristics shaped by adaptation to constant environmental conditions (Liang et al., 2025). In addition, the similarity between ancient and extant dog humeri measurements may suggest that the fundamental proportions of the humerus have remained relatively stable through time, as found by other authors (Bennett et al., 2016; MacKinnon, 2010) and further support the observation that small‐sized breeds were already present during the Roman period (Harcourt, 1974) including in Lusitania (Pires et al., 2017). However, due to the limited preservation of archaeological material, further research with a larger sample is needed to confirm this apparent morphological continuity regarding this structure. Other chronological periods could be studied as well in the future.

STF's relative position in extant dog humeri was significantly closer to the lateral epicondyle (11.80 ± 3.34 mm) than to the medial epicondyle (17.96 ± 5.32 mm), and its distance to the trochlear margin (12.12 ± 3.50 mm) was greater than to the lateral epicondyle, but shorter than to the medial epicondyle (Figure 1). A similar spatial pattern of the STF was also observed in Roman‐era dogs and Iberian wolf population. Interestingly, in humans, when STF is present, it tends to occupy a more central position within the distal humerus, being located only 2–3 mm closer to the medial epicondyle than to the lateral (Mathew et al., 2016; Nayak et al., 2009; Ndou et al., 2013). Prior to this research, no comparative data were available across temporal or taxonomic groups, limiting our understanding of the significance of this genus‐specific variation, although it could be linked to the intrinsic differences in muscle and ligament disposition for inherently different movement amplitude.

When analyzing STF shape, we found that the classification system frequently used in human studies, based on the foramen's outline as round, oval, triangular, or irregular (Baker et al., 2022; Bokhari et al., 2018; Mathew et al., 2016) may be subjective, especially considering the frequent irregularity in the margins of the STF observed across the studied groups (Figure 2). Therefore, and although at the risk of sacrificing the specific categorization of the few profoundly irregular shapes, we proposed a more precise approach by assessing the ratio between STF‐LL and STF‐TL, allowing a more consistent STF shape classification. The results revealed that STFs were predominantly elongated along the transverse axis of the distal epiphysis (ratio <0.95) or near‐equally proportioned (ratio 0.95–1.05), while more longitudinal shapes were less frequent across all three populations (Table 5). Interestingly, studies on human humeri have also reported that the STF is most commonly oval and elongated (Erdogmus et al., 2014; Mathew et al., 2016; Nayak et al., 2009; Pires et al., 2019; Singhal & Rao, 2007). Our findings provide new insights into the morphological variability of the STF in the *Canis* genus and emphasize the need for further studies to understand the implications of these variations.

The analysis of STF size revealed that extant dogs presented the smallest mean dimensions of the STF (STF‐LL: 5.12 ± 2.50 mm; STF‐TL: 6.00 ± 2.66 mm), compared to their wild counterpart or ancient dogs (STF‐LL: 6.02 ± 2.35 mm; STF‐TL: 7.04 ± 2.38 mm). Not surprisingly, this difference was even more apparent when compared to the Iberian wolf (STF‐LL: 9.90 ± 1.94 mm; STF‐TL: 10.73 ± 1.55 mm). The smaller size of the STF in extant dogs may be influenced by the overall reduction in humeral dimensions observed in our study, reflecting a reduction in body size within present‐day canine populations. As previously mentioned, this reduction has been linked to domestication processes and to the heterochrony shifts observed in skull and potentially other morphological traits (Evin et al., 2025; Geiger et al., 2017; Geiger et al., 2021).

Consistent with previous studies (Lamb, 1890), the present study reinforces that the presence of the STF is variable in both domestic dogs and Iberian wolves. However, it was notably less frequent in dogs. Specifically, 26.83% of extant dogs lacked this perforation, and the absence of the STF was more common in extant dogs compared to their Roman‐era ancestors (17.11%), and significantly more so than in the Iberian wolf population (1.43%).

In addition, more recent literature suggests that STF absence may be more common in smaller sized dogs, although detailed data on this correlation remains limited (Barone, 2020; Sisson & Grossman, 1977). Statistical analysis in this study evidenced that the STF in dogs was significantly less frequent in smaller humeri and more frequent in larger humeri. Furthermore, we found a strong positive correlation between overall humeral dimensions and STF size, suggesting that larger humeri are more likely to exhibit not only the presence of the STF but also proportionally larger foramina. Specifically, significant positive correlations were found between STF size and total humeral length, diaphyseal circumference, and epicondylar distance. These results support the notion that dog humeral size is a key determinant of this anatomical variation in dogs.

While the etiology of STF remains a subject of ongoing debate, Lamb (1890) and Chagas et al. (2016), offer an explanation based on the feeding posture of dogs adopted to tear the flesh of its prey, which may subject the distal humerus to increased mechanical pressure and explain the higher incidence of the STF in dogs compared to humans. However, Lamb's (1890) observation that canine elbow extension would be impossible without this foramen contradicts our findings. On the other hand, the hypothesis that bone robusticity may influence its development (Baker et al., 2022; Benfer & McKern, 1966; Myszka, 2015; Ndou & Schepartz, 2016) aligns with our own findings in dogs. However, it contrasts with Benfer and Tappen (1968) report, who proposed that the STF is less likely to occur in individuals with larger, more robust humeri. Even so, this hypothesis does not account for the variation in STF prevalence relative to the humeral size of dogs. Therefore, additional studies are needed to further investigate the underlying developmental or mechanical factors contributing to the formation of the STF in dogs.

## 5 CONCLUSIONS

This study represents a contribution to the assessment of STF in the domestic dog and the Iberian wolf. Our results demonstrate that the STF is an inconstant but frequent anatomical feature in *Canis lupus* subspecies, with a higher prevalence in the Iberian wolf compared to ancient and extant dogs. These findings suggest a gradual reduction in STF prevalence over time within domestic lineages, potentially linked to human‐driven selection processes and the resultant morphological diversity in dog breeds.

Osteometric analysis revealed that, unlike domestic dogs, Iberian wolves maintain relatively uniform humeral morphology, reinforcing the notion that selective breeding in domestic dogs has led to significant skeletal variability. Despite these differences, the relative positioning of the STF remained consistent across species, typically closer to the lateral epicondyle, which may reflect conserved developmental or biomechanical constraints.

Given the potential for the STF to influence the biomechanical properties of the distal humerus and its implications in diagnostic imaging and surgical procedures, its anatomical variability must be considered in clinical practice, and future research should further explore the genetic and developmental mechanisms underlying STF formation.

## AUTHOR CONTRIBUTIONS

M.B., L.M‐J., A.E.P., and J.C. carried out study design. M.B., M.S., E.M., D.F., A.E.P., and J.C. conducted data processing, data analysis, results interpretation, and manuscript drafting. L.M‐J., G. A‐P., C.D., S.G., D.F., and A.E.P. provided access to bone specimens. A.E.P and J.C. managed project conception, funding acquisition, and critical revision of the manuscript. All authors read, provided feedback, and approved the manuscript.

## Data Availability

The data that support the findings of this study are available from the corresponding author upon reasonable request.
